# Effect of rifampin and itraconazole on the pharmacokinetics of zanubrutinib (a Bruton's tyrosine kinase inhibitor) in Asian and non-Asian healthy subjects

**DOI:** 10.1007/s00280-019-04015-w

**Published:** 2019-12-26

**Authors:** Song Mu, Zhiyu Tang, William Novotny, Manal Tawashi, Ta-Kai Li, Ying Ou, Srikumar Sahasranaman

**Affiliations:** 1 Clinical Pharmacology, BeiGene USA, 2955 Campus Drive, Suite 300, San Mateo, CA 94403 USA; 2 Clinical Development, BeiGene USA, San Mateo, CA USA; 3 Clinical Operations, BeiGene USA, San Mateo, CA USA; 4 Drug Safety and Pharmacovigilance, BeiGene USA, San Mateo, CA USA

**Keywords:** Clinical pharmacology, Clinical trials, Drug–drug interactions, Oncology, Pharmacokinetics and drug metabolism

## Abstract

**Purpose:**

Zanubrutinib (BGB-3111) is a potent Bruton’s tyrosine kinase inhibitor with promising clinical activity in B-cell malignancies. Zanubrutinib was shown to be mainly metabolized through cytochrome P450 3A (CYP3A) in vitro. We evaluated the effect of steady-state rifampin (a strong CYP3A inducer) and steady-state itraconazole (a strong CYP3A inhibitor) on the pharmacokinetics (PK), safety, and tolerability of zanubrutinib in healthy Asian and non-Asian subjects.

**Methods:**

In this open-label, two-part clinical study, 20 participants received a single oral dose of zanubrutinib (320 mg) and oral rifampin (600 mg) in Part A, and 18 participants received a single oral dose of zanubrutinib (20 mg) and oral itraconazole (200 mg) in Part B. Serial blood samples were collected after administration of zanubrutinib alone and zanubrutinib in combination with rifampin or itraconazole for the measurement of PK parameters.

**Results:**

Coadministration with rifampin decreased AUC_0–∞_ of zanubrutinib by 13.5-fold and *C*_max_ by 12.6-fold. Coadministration with itraconazole increased the AUC_0–∞_ of zanubrutinib by 3.8-fold and *C*_max_ by 2.6-fold. The PK of zanubrutinib was consistent between Asian and non-Asian subjects, and  zanubrutinib was well tolerated in this study.

**Conclusions:**

These results confirm that zanubrutinib is primarily metabolized by CYP3A in humans. The PK of zanubrutinib was comparable between Asian and non-Asian subjects and, therefore, no dose modifications are necessary for zanubrutinib in these ethnic populations.

**Electronic supplementary material:**

The online version of this article (10.1007/s00280-019-04015-w) contains supplementary material, which is available to authorized users.

## Introduction

The B-cell receptor signaling pathway is essential for normal B-cell development but is also implicated in the survival and proliferation of malignant B cells [1–4]. Inhibition of B-cell receptor signaling has recently been established as an effective approach for the management of B-cell malignancies [2]. Bruton’s tyrosine kinase (BTK) is a key component of the B-cell receptor signaling pathway [3], and the first-generation BTK inhibitor, ibrutinib, has become a standard of care in chronic lymphocytic leukemia/small lymphocytic lymphoma (CLL/SLL), mantle cell lymphoma, and Waldenström macroglobulinemia [5–13].

Zanubrutinib (BGB-3111) is an investigational, highly specific, next-generation BTK inhibitor that has demonstrated encouraging clinical activity in phase 1/2 studies [14–17] and is currently in phase 3 testing for multiple indications [18–20]. In patients with B-cell malignancies, zanubrutinib was generally well tolerated at total daily doses ranging from 40 to 320 mg, and the recommended phase 2 dose is 160 mg administered twice daily [16]. Pharmacokinetic (PK) data showed that zanubrutinib was rapidly absorbed after oral administration, with *C*_max_ observed at approximately 2 h after dosing in patients with B-cell malignancies. Exposure to zanubrutinib increased in a dose-proportional manner from total daily doses of 40–320 mg and the mean half-life of zanubrutinib was approximately 2–4 h with minimal accumulation observed after repeated dosing. Total exposure was comparable when 320 mg total daily dose was administered as 160 mg twice daily or 320 mg once daily [21]. In human liver microsomes, the oxidative metabolism of zanubrutinib is largely mediated through the cytochrome P450 3A (CYP3A) pathway (unpublished data).

Drug–drug interactions (DDI) with CYP3A-mediated metabolism are particularly problematic in patients with B-cell malignancies such as CLL/SLL because these patients are at risk for systemic fungal infections due to underlying disease-related immune dysfunction and therapy-related immunosuppression [22]. Prophylactic or therapeutic use of azole anti-fungals is common in CLL/SLL [23], and the agents in this therapeutic class (e.g., voriconazole, posaconazole) are moderate-to-strong inhibitors of CYP3A [24, 25]. Because of the potential concomitant usage of these agents with zanubrutinib, it is important to understand the DDI potential between zanubrutinib and CYP3A inhibitors and inducers. Hence, the current study was designed to evaluate the effect of rifampin (a strong CYP3A inducer) and itraconazole (a strong CYP3A inhibitor) on the PK of zanubrutinib in healthy subjects. Rifampin and itraconazole were chosen because they are a preferred CYP3A inducer and inhibitor, respectively, in DDI studies [26, 27].

Due to the anticipated PK variability for zanubrutinib as a CYP3A substrate and underlying comorbidities in cancer patients, PK comparisons across patient studies to understand potential ethnic differences could be challenging. To assess ethnic differences in PK more robustly, it is ideal to use a single protocol that controls extrinsic factors and that can be uniformly applied to the different ethnic populations. To this end, this DDI study included healthy Asian and non-Asian subjects to facilitate comparison of zanubrutinib PK across different ethnic groups and to help support dose selection of zanubrutinib in clinical studies conducted in Asia.

## Methods

### Study design and subjects

The study was conducted in accordance with the International Conference on Harmonisation, Good Clinical Practice, and the Declaration of Helsinki guidelines. All participants provided written informed consent prior to study entry. The protocol was reviewed and approved by the Institutional Review Board at the study center.

This was an open-label, parallel-group, fixed-sequence study in healthy male and female subjects conducted in two parts, Part A and Part B. Part A investigated the effect of CYP3A induction by steady-state rifampin on the single-dose PK of 320 mg zanubrutinib, and Part B investigated the effect of CYP3A inhibition by steady-state itraconazole on the single-dose PK of 20 mg zanubrutinib.

Healthy male or female subjects, aged between 18 and 65 years, inclusive, with a body mass index between 18.0 and 32.0 kg/m^2^, inclusive, who were of either first- or second-generation Asian descent (defined as an individual whose biological parents or four biological grandparents were born in one of the following East Asian countries or territories: China, Japan, Korea, Taiwan, Hong Kong, Mongolia, Cambodia, or Vietnam); or non-Asian descent (defined as an individual whose biological parents and four biological grandparents were not born in one of the previously listed East Asian countries), were selected according to the inclusion and exclusion criteria listed in the protocol.

It was planned that approximately 40 subjects (20 in each part) would be enrolled to ensure that at least 36 subjects (18 in each part) completed the study. It was planned that approximately one-third of subjects enrolled in the study would be Asian, and approximately two-thirds would be non-Asian.

### Treatments

In Part A, participants received a single oral dose of zanubrutinib (320 mg) in the fasted state on Day 1 and Day 10. Oral rifampin (600 mg/day, Rifampin, VersaPharm Inc., Marietta, GA, USA) was administered once daily in the fasted state on Days 3 through 11. In Part B, participants received a single oral dose of zanubrutinib (20 mg) in the fasted state on Day 1 and Day 6. Oral itraconazole (200 mg/day, Sporanox® capsules, Janssen Pharmaceuticals, Titusville, NJ, USA) was administered once-daily approximately 30 min after completing a meal on Days 3, 4, 5, and 7, and in the fasted state on Day 6.

### Study assessments

#### Sample collection

Serial blood samples were collected with zanubrutinib alone and when combined with rifampin or itraconazole for the measurement of zanubrutinib plasma concentrations. Plasma samples were collected at predose, 0.5, 1, 1.5, 2, 3, 4, 6, 8, 12, 24, 36, and 48 h postdose on Day 1 and Day 10 in Part A and on Day 1 and Day 6 in Part B.

#### Bioanalytical methodology

Plasma concentrations of zanubrutinib were determined using validated high-performance liquid chromatography coupled with tandem mass spectrometry (HPLC–MS/MS) (Xenobiotic Laboratories, NJ, USA). Protein precipitation was utilized to extract the analyte and internal standard from human plasma containing dipotassium ethylenediaminetetraacetic acid (K2EDTA) as anticoagulant. The analytical method utilized a reversed-phase HPLC column (Supelco Ascentis Express C18 20 × 2.1 mm, 2.7 µm) with a gradient flow of 0.1% formic acid in water (Mobile Phase A) and methanol:acetonitrile (1:1) (Mobile Phase B) at a rate of 0.5 mL/min. The analyte and internal standard were detected using an AB Sciex API-4000 LC–MS/MS system equipped with a positive ESI ion detection.

The calibration range of zanubrutinib in the assay was 1.00 to 1000 ng/mL using a 50 μL aliquot of plasma, with the lower limit of quantification of 1.00 ng/mL. The performance of calibration standards showed a good linearity from 1.00 to 1000 ng, with the coefficient of correlation (*r*) > 0.990, and the cumulative bias ranged from − 2.00 to 3.00% and the cumulative precision was ≤ 5.34% coefficient of variation (CV). The results indicate the method to be sensitive, selective, accurate, and reproducible.

#### Pharmacokinetic analysis

Noncompartmental PK analysis was conducted using the Phoenix^®^ WinNolin^®^ software (Version 6.4). PK parameters were derived for zanubrutinib alone and in combination with rifampin or itraconazole, including area under the plasma concentration–time curve (AUC), maximum observed plasma concentration (*C*_max_), time of the maximum observed plasma concentration (*t*_max_), apparent terminal elimination half-life (*t*_1/2_), apparent total oral clearance (CL/*F*), and apparent volume of distribution during the terminal elimination phase (*V*_z_/*F*). Log-transformed area under the curve from time 0 extrapolated to time *t* (AUC_0–*t*_), area under the curve from time 0 extrapolated to infinite time (AUC_0–∞_), and *C*_max_ of zanubrutinib were analyzed using a mixed model for each part of the study, including treatments as a fixed effect and subject as a random effect. Estimates of geometric mean ratios (GMRs) and the corresponding 90% confidence intervals (CIs) were derived for the comparisons of AUC_0–∞_, and *C*_max_ as follows:

Part A: zanubrutinib coadministration with rifampin (test) versus zanubrutinib alone (reference),

Part B: zanubrutinib coadministration with itraconazole (test) versus zanubrutinib alone (reference).

### Safety analysis

Treatment-emergent adverse events (TEAEs) are summarized by part, treatment, National Cancer Institute Common Terminology Criteria for Adverse Events (v4.03) grade, and relationship to study drugs (zanubrutinib, rifampin, and itraconazole). The frequency of TEAEs is summarized by part, treatment, and Medical Dictionary for Regulatory Activities (MedDRA) system organ class (SOC) and preferred term. The summary and frequency TEAE tables are presented for all causalities and for those TEAEs considered related to the study drugs.

## Results

### Demographics

In Part A, 20 subjects were enrolled, and 19 subjects completed the study; in Part B, 18 subjects were enrolled, and 17 subjects completed the study. All subjects in Parts A and B were included in the safety and PK analysis sets. Baseline demographics for participants in Part A and Part B are shown in Table 1. The mean participant age was 40 years, and 76.3% were male. In Part A and Part B, 40.0% and 44.4%, respectively, were Asian. All Asian patients included in the study were of first-generation Chinese descent.

**Table 1:** Summary of participant demographics at screening

	Part A (*N* = 20)	Part B (*N* = 18)	Overall (*N* = 38)
Age, years			
Mean (SD)	39 (9.6)	42 (10.6)	40 (10.1)
Sex, *n* (%)			
Male	15 (75.0)	14 (77.8)	29 (76.3)
Female	5 (25.0)	4 (22.2)	9 (23.7)
Race, *n* (%)			
Asian	8 (40.0)	8 (44.4)	16 (42.1)
Black or African American	4 (20.0)	1 (5.6)	5 (13.2)
White	7 (35.0)	9 (50.0)	16 (42.1)
Multiple	1 (5.0)	0	1 (2.6)
Ethnicity			
Hispanic or Latino	2 (10.0)	3 (16.7)	5 (13.2)
Not Hispanic or Latino	18 (90.0)	15 (83.3)	33 (86.8)
Weight, kg			
Mean (SD)	76.6 (8.75)	81.0 (15.6)	78.6 (12.5)
Height, cm			
Mean (SD)	173 (8.5)	174 (12.7)	174 (10.6)
BMI, kg/m^3^			
Mean (SD)	25.5 (1.73)	26.5 (2.93)	26.0 (2.39)

*BMI* body mass index, *N* number of subjects, *QD* once daily, *SD* standard deviation

Part A, Day 1: single oral dose of 320 mg zanubrutinib; Days 3 to 9 and 11: oral dose of 600 mg rifampin QD. Day 10: single oral dose of 320 mg zanubrutinib coadministered with 600 mg rifampin QD

Part B, Day 1: single oral dose of 20 mg zanubrutinib; Days 3 to 5 and 7: oral dose of 200 mg itraconazole QD

### Pharmacokinetics

#### Part A

**Fig. 1:**
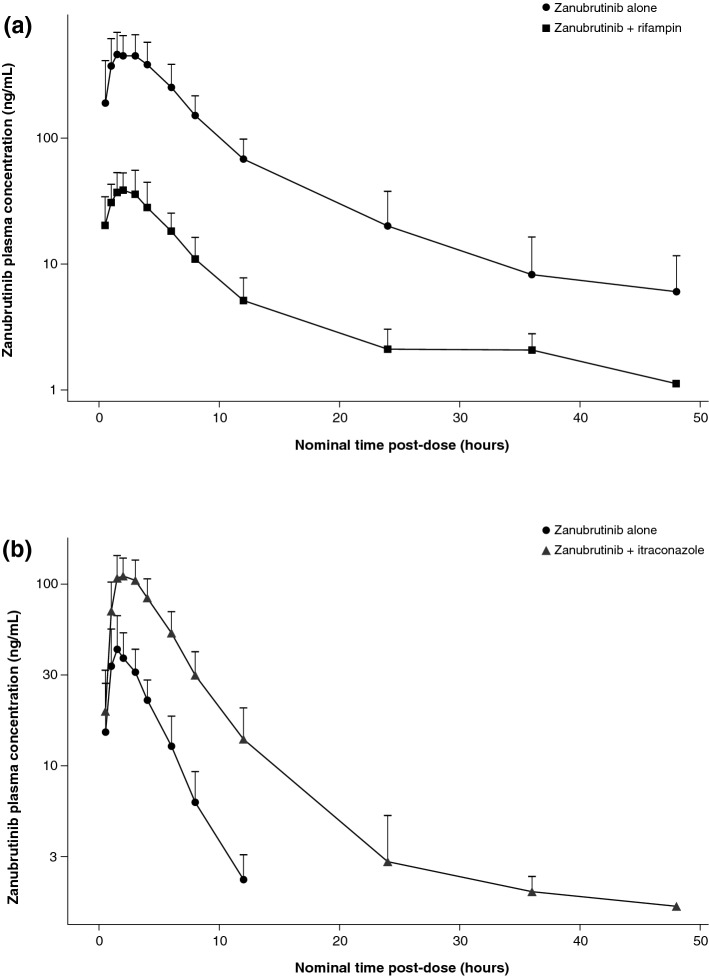
Arithmetic mean (+ SD) zanubrutinib plasma concentration profiles following **a** administration of 320 mg alone and coadministration with 600 mg rifampin or **b** administration of 20 mg alone and coadministration with 200 mg itraconazole. Zanubrutinib plasma concentrations on *Y*-axis are shown on log scale

The plasma concentration–time profiles and PK parameters of zanubrutinib in the absence and presence of rifampin are shown in Fig. 1a and Table 2, respectively. Plasma concentrations of zanubrutinib were significantly lower following coadministration of 320 mg zanubrutinib with 600 mg rifampin compared with the administration of 320 mg zanubrutinib alone. As shown in Table 2, GMRs (90% CI) of AUC_0–∞_ and *C*_max_ for zanubrutinib were 7.4% (6.0–9.1) and 7.9% (6.6–9.5), respectively. These results represented a decreased exposure of 13.5-fold for AUC_0–∞_, and 12.6-fold for *C*_max_ when zanubrutinib was co-administered with rifampin. The geometric mean apparent *t*_1/2_ of zanubrutinib was shorter following coadministration of rifampin (4.8 h) compared to administration of zanubrutinib alone (6.8 h). AUC_0–*t*_ values were consistent with AUC_0–∞_ values because of the short half-life of zanubrutinib, and hence only AUC_0–∞_ values are presented in this manuscript. Apparent clearance (CL/*F*) was increased from 93 to 1249 L/h, and this likely reflects a significant decrease in bioavailability (*F*) of zanubrutinib when coadministered with rifampin.

**Table 2:** Summary of pharmacokinetic parameters of zanubrutinib following administration of 320 mg zanubrutinib alone and coadministration with 600 mg rifampin – Part A

Pharmacokinetic parameter (units)^a^	320 mg zanubrutinib (*n* = 20)	320 mg zanubrutinib + 600 mg rifampin QD (*n* = 20)	Geometric ratio of adjusted means, % (90% CI)^c^
AUC_0–∞_, h·ng/mL (mean CV%)	3524 (36) (*n* = 18)	261 (43) (*n* = 18)	7.4 (6.0, 9.1)
*C*_max_, ng/mL	532 (40)	42 (41)	7.9 (6.6, 9.5)
*t*_max_^b^, h	2.0 (0.5–6.0)	2.0 (0.5–4.0)	–
*t*_1/2_, h	6.8 (54)	4.8 (91)	–
CL/*F*, L/h	93 (36)	1249 (43)	–
*V*_z/_F, L	914 (73)	8665 (70)	–

*AUC* area under the plasma concentration–time curve, *CL/F* apparent total oral clearance, *C*_*max*_ maximum plasma concentration, *QD* once daily, *t*_*1/2*_ apparent terminal elimination half-life, *t*_*max*_ time of the maximum observed plasma concentration, *V*_*z*_*/F* apparent volume of distribution during the terminal elimination phase

^a^Geometric mean data (% coefficient of variation) except where otherwise noted

^b^Median (min–max)

^c^Ratio of zanubrutinib in combination with rifampin versus zanubrutinib alone

**Fig. 2:**
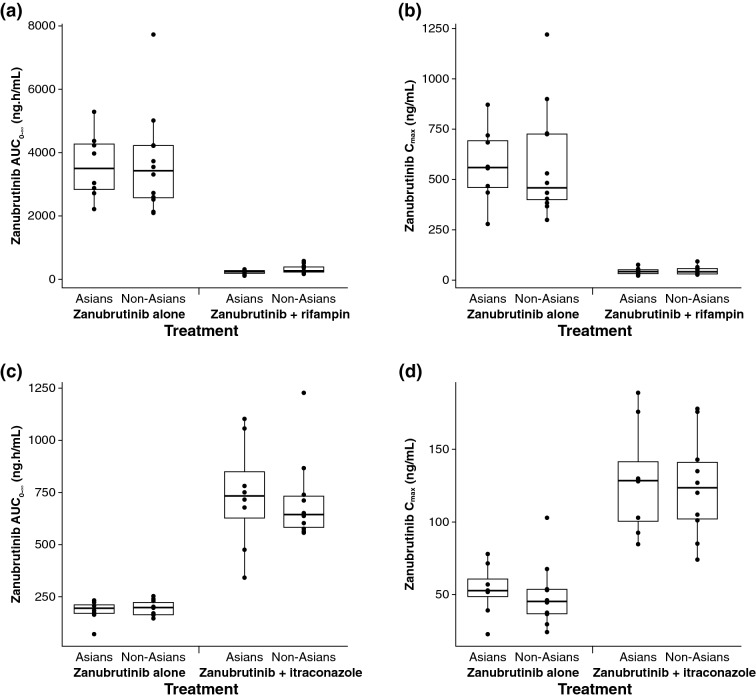
Comparative box plots of area under the plasma concentration–time curve from 0 h to infinity (AUC_0–∞_, ng·h/mL) and maximal plasma concentration (*C*_max_; ng/mL) in Asian and non-Asian) subjects in **a**, **b** the absence and presence of rifampin and **c**, **d** in the absence and presence of itraconazole. The box plot represents 25th and 75th percentiles; whiskers extend to 5th and 95th percentiles. Median is indicated by a line within the box, and circles represent values for individual subject

The PK parameters of zanubrutinib were comparable between Asian and non-Asian subjects following administration of 320 mg zanubrutinib alone on Day 1 as well as coadministration with 600 mg rifampin on Day 10 (Supplementary Table 1 and Fig. 2a, b).

## Part B

The plasma concentration–time profiles and PK parameters of zanubrutinib in the absence and presence of itraconazole are shown in Fig. 1b and Table 3, respectively. Plasma concentrations of zanubrutinib were significantly higher following coadministration of 20 mg zanubrutinib with 200 mg itraconazole than the administration of 20 mg zanubrutinib alone. As shown in Table 3, GMRs (90% CI) for AUC_0–∞_ for zanubrutinib was 378% (344–415), respectively. The GMR (90% CI) for zanubrutinib *C*_max_ was 257% (226–291). These results represented an increased exposure of 3.8-fold for AUC_0–∞_, and 2.6-fold for *C*_max_ when zanubrutinib was co-administered with itraconazole. The geometric mean apparent t_1/2_ of zanubrutinib was longer following coadministration of 20 mg zanubrutinib with 200 mg itraconazole (4.3 h) compared to administration of 20 mg zanubrutinib alone (2.2 h). However, as the terminal elimination phase of zanubrutinib may not have been adequately characterized with the 20-mg dose (due to concentrations being below the limit of quantification beyond 12 h post-dose), the increase in t_1/2_ following co-administration with itraconazole should be interpreted with caution.

**Table 3:** Summary of pharmacokinetic parameters of zanubrutinib following administration of 20 mg zanubrutinib alone and coadministration with 200 mg itraconazole – Part B

Pharmacokinetic parameter (units)^a^	20 mg zanubrutinib (*n* = 18)	20 mg zanubrutinib + 200 mg itraconazole QD (*n* = 18)	Geometric ratio of adjusted means, % (90% CI)^c^
AUC_0–∞_, h·ng/mL	184 (29)	693 (31)	378 (344, 415)
*C*_max_, ng/mL	48 (41)	122 (29)	257 (226, 291)
*t*_max_^b^, h	1.5 (1.0–4.0)	2.0 (1.0–3.0)	–
*t*_1/2_, h	2.2 (18.2)	4.3 (45)	–
CL/*F*, L/h	109 (29)	29 (31)	–
*V*_z/_*F*, L	341 (34)	178 (29)	–

*AUC* area under the plasma concentration–time curve, *CL/F* apparent total oral clearance, *C*_*max*_ maximum plasma concentration, *QD* once daily, *t*_*1/2*_ apparent terminal elimination half-life, *t*_*max*_ time of the maximum observed plasma concentration, *V*_*z*_*/F* apparent volume of distribution during the terminal elimination phase

^a^Geometric mean data (% coefficient of variation) except where otherwise noted

^b^Median (min–max)

^c^Ratio of zanubrutinib in combination with itraconazole versus zanubrutinib alone

The PK of zanubrutinib was comparable between Asian and non-Asian subjects following administration of 20 mg zanubrutinib alone on Day 1 and coadministration with 200 mg itraconazole on Day 6 (Supplementary Table 1 and Fig. 2c, d).

### Safety

Zanubrutinib was well tolerated in this study. The overall incidence of TEAEs was low–less than 30% in both Part A and Part B. Single doses of 320 mg and 20 mg zanubrutinib administered alone or co-administered with 600 mg rifampin and 200 mg itraconazole, respectively, were well tolerated in healthy subjects. In both parts, no subject reported a TEAE higher than Grade 2 or an SAE, and no subject discontinued due to a TEAE. The majority of TEAEs were considered not related to the study drugs, were Grade 1 in severity, and resolved without treatment. No clinically significant changes or findings were noted in clinical laboratory evaluations, vital signs, physical examinations, or body weight in this study. No subject had a QTcF value > 450 ms or an increase from baseline in QTcF of > 60 ms during the study.

## Discussion

The results from this clinical assessment confirm that zanubrutinib is primarily metabolized by CYP3A in humans and is a sensitive CYP3A substrate. Rifampin significantly affected the bioavailability and apparent clearance of zanubrutinib as reflected by a 13.5-fold decrease in AUC_0–∞_, 12.6-fold decrease in *C*_max_ when co-administered with rifampin. Therefore, zanubrutinib should not be co-administered with strong CYP3A inducers such as rifampin as the resulting decrease in zanubrutinib exposure may impact its efficacy.

Itraconazole increased the bioavailability and decreased the apparent clearance of zanubrutinib, as evident by the increased exposure of 3.8-fold for AUC_0–∞_, and 2.6-fold for *C*_max_. DDI with strong CYP3A inducers and inhibitors have also been reported for other BTK inhibitors such as ibrutinib (24-fold increase in AUC with ketoconazole and tenfold reduction in AUC with rifampin) and acalabrutinib (5.1-fold increase in AUC with itraconazole and 77% decrease in AUC with rifampin) [25, 28].

The absolute bioavailability of zanubrutinib is unknown and, therefore, based on the magnitude of interaction observed in Part A of this study and the known interactions between CYP3A inhibitors and other BTK inhibitors, a dose of 20 mg of zanubrutinib was conservatively chosen for Part B to study the CYP3A inhibition with itraconazole. Although the 20 mg dose was not studied previously, based on the available PK data from this study and other studies [21], zanubrutinib exhibits dose-proportional and linear PK over the dose range of 20–320 mg. Therefore, the results from the 20 mg dose used in this study can be extrapolated to clinically relevant doses such as 160 mg BID.

The half-life after administration of the 320 mg dose without rifampin was 6.8 h, which was longer than the 2–4 h half-life observed in previous studies in patients with B-cell malignancies. The difference may be attributed to the different PK sampling scheme used in this study where PK was followed until 48 h post-dose. In patient studies, PK was generally assessed only for 8 h post-dose [21] to improve patient convenience, and the half-life may have been underestimated in patients.

Asian and non-Asian healthy subjects were enrolled in this dedicated DDI study to assess any ethnic differences in PK and to support dose selection and clinical development of zanubrutinib in Asia. The exposure of zanubrutinib in Asian subjects was comparable with the non-Asian subjects at both 20-mg and 320-mg doses, and the magnitude of interaction with rifampin and itraconazole was also comparable between the Asian and non-Asian subjects. These results confirm that there are no significant ethnic differences in PK of zanubrutinib, and dose-adjustments are not required in Asian patients. The current study provides a reliable assessment of ethnic comparisons of PK as part of a dedicated clinical pharmacology study in healthy subjects when cross-study comparisons can be challenging in patients because of the variability in PK and underlying comorbidities for a sensitive CYP3A substrate.

Fungal and bacterial infections are commonly observed in patients with B-cell malignancies and may require treatment with medications that are strong or moderate inhibitors of CYP3A such as voriconazole and posaconazole. In these instances of co-medication with CYP3A inhibitors, it would be ideal for patients to continue BTK inhibitor therapy, because it has been demonstrated that dose interruptions with BTK inhibitors can affect their efficacy. For example, patients with chronic lymphocytic leukemia/small lymphocytic lymphoma missing ≥ 8 consecutive days of ibrutinib had a shorter progression-free survival compared with those missing < 8 days [29]. Based on the magnitude of interaction with the strong CYP3A inhibitor itraconazole in this study (< 4-fold increase in AUC), it is anticipated that patients taking zanubrutinib may not require dose interruptions and could continue treatment with a lower dose when coadministered with a strong inhibitor of CYP3A to avoid exceeding exposures associated with the maximum clinically tested dose of 320 mg daily.

The results from this DDI study will be used to refine existing in silico physiologically-based PK (PBPK) simulations (unpublished) and assess the impact of moderate and weak CYP3A inhibitors and inducers on the PK of zanubrutinib. Findings from this clinical study and the PBPK simulations in conjunction with safety and efficacy data from clinical studies will be used to recommend appropriate dose modifications when patients are required to take zanubrutinib concomitantly with moderate or strong inhibitors and inducers of CYP3A.

Single doses of 320 mg and 20 mg zanubrutinib administered alone or co-administered with 600 mg rifampin and 200 mg itraconazole, respectively, were well tolerated in healthy subjects in this study.

## Conclusions

The results from this DDI study confirm that zanubrutinib is primarily metabolized by CYP3A in humans. PK of zanubrutinib was comparable between Asian and non-Asian subjects and, therefore, no dose modifications are necessary for zanubrutinib in these ethnic populations.

## Electronic supplementary material

Below is the link to the electronic supplementary material.

Supplementary file1 (DOCX 33 kb)
