# Supplementary material for: Effect of rifampin and itraconazole on the pharmacokinetics of zanubrutinib (a Bruton's tyrosine kinase inhibitor) in Asian and non-Asian healthy subjects
Source: Cancer Chemother Pharmacol. 2019 Dec 26;85(2):391–9. doi: 10.1007/s00280-019-04015-w (PMC7015960; doi:10.1007/s00280-019-04015-w)
Supplement: Supplementary file 1 — Supplementary file1 (DOCX 33 kb) [file 280_2019_4015_MOESM1_ESM.docx]

**Supplementary Materials**

**Effect of rifampin and itraconazole on the pharmacokinetics of zanubrutinib (a Bruton’s tyrosine kinase inhibitor) in Asian and non-Asian healthy subjects**

Song Mu^1^ • Zhiyu Tang^1^ • William Novotny^2^ • Manal Tawashi^3^ • Ta-Kai Li^4^ • Ying Ou^1^ • Srikumar Sahasranaman^1^

^1^ Clinical Pharmacology, BeiGene USA, San Mateo, CA

^2^ Clinical Development, BeiGene USA, San Mateo, CA

^3^ Clinical Operations, BeiGene USA, San Mateo, CA

^4^ Drug Safety and Pharmacovigilance, BeiGene USA, San Mateo, CA

**Concise title:** Effect of rifampin and itraconazole on zanubrutinib pharmacokinetics

Corresponding author and address:

Srikumar Sahasranaman, PhD

2955 Campus Drive, Suite 400

San Mateo, CA 94403

USA

Tel: +1-831-232-8732

E-mail: sri.sahasranaman@beigene.com

Supplementary Table 1 Pharmacokinetics of zanubrutinib in Asian and non-Asian subjects following administration of 320 mg zanubrutinib alone on Day 1 and coadministration with 600 mg rifampin on Day 10 (Part A) or administration of 20 mg zanubrutinib alone on Day 1 and coadministration with 200 mg itraconazole on Day 6 (Part B)

| **Pharmacokinetic parameters^a^, units** | **Asian subjects** | **Non-Asian subjects** |
| --- | --- | --- |
| **Part A** | n=8 | n=12 |
| **Day 1 (zanubrutinib alone)** | | |
| AUC_0-∞,_ h*ng/mL | 3459 (30) | 3411 (42) (n=11) |
| C_max_, ng/mL | 543 (36) | 525 (44) |
| **Day 10 (zanubrutinib + rifampicin)** | | |
| AUC_0-∞_, h*ng/mL | 213 (38) | 293 (43) |
| C_max_, ng/mL | 41 (41) | 43 (43) |
| **Part B** | n=8 | n=10 |
| **Day 1 (zanubrutinib alone)** | | |
| AUC_0-∞_, h*ng/mL | 174 (40) | 192 (20) |
| C_max_, ng/mL | 50 (40) | 46 (44) |
| **Day 6 (zanubrutinib + itraconazole)** | | |
| AUC_0-∞_, h*ng/mL | 694 (40) | 693 (25) |
| C_max_, ng/mL | 124 (29) | 120 (30) |

*AUC*, area under the plasma concentration-time curve, *C_max_*_,_ maximum plasma concentration.

^a^ represented as geometric means (% coefficient of variation) for AUC_0-∞_, and C_max_
